# Hospital and Institutional News

**Published:** 1916-06-03

**Authors:** 


### HOSPITAL AND INSTITUTIONAL NEWS.

### SIR DOUGLAS HAIG'S DISPATCHES.

Sir Douglas Haig's first dispatches, dealing with the operations on the Western Front from December 15, 1915, to May 15, 1916, contain a paragraph of special commendation concerning the Army Medical Services; and it will be noticed that the civil medical practitioners serving in the Royal Army Medical Corps receive for the first time corporate recognition of the work they are doing. A new feature of this dispatch is the mentioning of individual units for gallant and good services: no medical unit appears in the list, and as long as immobile trench war goes on it is perhaps unlikely that any medical unit will be so distinguished. Certainly if professional excellence among the lines of communication units (casualty clearing stations, general hospitals, stationary hospitals, ambulance trains) were to be recognised in dispatches, the selection of units for mention would become a difficult and invidious task; for the general level of work and efficiency is exceedingly high. And the same holds good to a great extent of the field ambulances and sanitary sections, which form integral parts of the Divisions at the Front.

### PREVENTION OF SICKNESS RECOGNISED.

Like the up-to-date commander that he is, Sir Douglas recognises and emphasises the value of the prevention of Army sickness. He says that "the sick-rate has been consistently low; there has been no serious epidemic, and enteric fever, the bane of armies in the past, has almost completely disappeared owing to preventive measures energetically carried out." Trench-foot, also, has been nearly eliminated, or at least kept in check, by careful application of the precautions recommended and taught by regimental medical officers. Then follows the tribute, already mentioned, to "those members of the civil medical profession who have patriotically given their valuable services to the Army"; and this section of the dispatch closes with a tribute to the marked efficiency and devotion to duty of the members of the Canadian Army Medical Corps.

### WOMEN AND CAMBRIDGE UNIVERSITY MEDICAL EXAMINATIONS.

A new "grace" is being brought forward at Cambridge University to allow women to sit for the first and second examinations for the M.B. degree. A certain amount of opposition is developing to the proposal, partly, at least, on account of alleged irregularities in the way in which the new "grace" is being proposed. It remains, therefore, to be seen whether this small instalment of increase in women's opportunities will be granted or not. The scheme does not provide that women may take the Cambridge degrees, nor that they may sit for the final (3rd) M.B. examinations: it is confined to the examinations in the preliminary sciences and in anatomy and physiology. It is stated in support of the proposal that women who go to Cambridge to study natural science and at the same time intend to take up medicine ultimately are handicapped at present, as compared with men, in that their three years at the University does not count towards their medical curriculum, which has to be completed separately afterwards; whereas, if this "grace" is carried, they will in future stand on an equality with men in this respect (except that they are not allowed to possess the University degree).

### THE WOUNDED AND THE LONDON SQUARES.

The correspondence which has been occupying the daily newspapers on the throwing open of London squares for the use of convalescent military patients will, we trust, result in an increase of such concessions on the part of householders and garden committees. There stand revealed in the letters which have been published some unaccountable instances of illiberality on the part of those who control gardens of this sort: one such is the conduct of the authorities of the Chelsea Physick Garden, a particularly flagrant case. There are also, it is pleasing to note, several instances where gardens or squares have already been spontaneously made available for soldiers. There is need, also, for a certain amount of discrimination and levelheadedness in this matter. It is by no means every garden square that is suitable for this purpose. The great desiderata are quiet, size, and freedom from dust and other annoyances. To give an instance, we confess to a good deal of sympathy with those residents in quite tiny squares within a stone's throw of Hyde Park or similar open spaces, who have had the courage to point out the unsuitability of a small strip of noisy, hot, dusty garden space when there is a large, quiet, cool park across the road.

### THE GENERAL MEDICAL COUNCIL.

The General Medical Council, which for a long time has aspired to offices more in keeping with the dignity of the directing authority of a great profession, met for the first time last week in its new home in Hallam Street, London. As the membership of the Council expanded with the addition of representatives of the newer universities, the old premises in Oxford Street became cramped, and more generous accommodation became imperative. The council chamber of the new building is a fine, well-lighted, oak and white apartment, both spacious and quiet. The president and the chairman of committees sit at a raised bench, the other members being provided with chairs and desks. To the portraits and busts of former presidents which covered the walls of the Oxford Street council chamber has been added another bust—that of the late Sir William Turner, the immediate predecessor of Sir Donald MacAlister, the present president. Sir William Turner's bust is placed behind the presidential chair, along with a bust of Sir Richard Quain.

### A MEDICAL SCHOOL FOR THE TRANSVAAL.

An important meeting has recently been held in Johannesburg to forward an ambitious scheme for the establishment in that city of a medical school for the Transvaal. The resolution adopted urges such a step, and proceeds to ask also for facilities for the granting of diplomas. Quite possibly this scheme may prove the precursor some day of a Transvaal University, a project which would surely appeal strongly to the sympathies of the Rhodes Trustees. Dr. Watkins-Pitchford, Director of the South African Institute for Medical Research, in his speech proposing the resolution, dwelt upon the fact that the population of Johannesburg and the adjacent villages along the line of the reef is about 430,000, of whom 206,000 are whites. The number of hospital beds serving this population is 8,170, and it is obvious that the wealth of clinical material there gathered together must be very great. It was decided to communicate the resolution (which was unanimously passed) to the Prime Minister (General Botha), the Minister for Education, and all members of Parliament.

### HOSPITAL AID FROM THE UNITED STATES.

An organisation has been set on foot in America entitled Aid for the Allied Hospitals. It is under the patronage of Colonel Roosevelt and of Mr. Herrick, late American Ambassador in Paris: the headquarters are in New York and branches are to be established in every State of the Union. The immediate object of this organisation is to obtain subscriptions for the hospitals of the Allied nations and to assist war victims, particularly in France and England. Besides those already mentioned, the leading members of the committee are: Mr. Westinghouse, chairman; Sir Arthur Herbert and Mr. J. S. Bache, treasurers; the British Ambassador (Sir Cecil Spring-Rice), and the Ambassadors of France, Russia, Italy, the Belgian Minister, and Sir Robert Borden, the Premier of Canada. The prevailing sentiment in America about this war is, we are thankful to know, almost wholly with the Allies; and any organisation of help which does not strain the letter or the spirit of neutrality is certain of enthusiastic support. This very practical form of help will arouse here and amongst our Allies both gratification and gratitude.

### NAVAL HOSPITAL STANDARD.

Mr. Fox, the chairman of the R.N.A. Hospital, Truro, has received an interesting letter from Sir Arthur May, Medical Director-General, from which we take the following:—"Dear Mr. Fox,—I had the pleasure of visiting and inspecting the Truro Naval Hospital. . . . The organisation was first rate, and I was much struck by the able way an old building had been converted into a really excellent hospital. The whole hospital was quite as clean as one of our first-class naval hospitals, and I can picture no higher standard. I appreciate to the full the valuable work and great help to the Navy that one and all working in and for the hospital are doing, and especially do I value the efforts of those ladies who are performing so efficiently the less showy work of the hospital." The last tribute to the valuable and unostentatious work of the ladies referred to is a very grateful one; for, like most necessary work, it is monotonous and mostly unexciting. Therefore, it is all the more worthy of official recognition and thanks. These ladies, too, will appreciate the favourable comparison drawn of the cleanliness of their own hospital with that of the Navy, which has become a tradition even among those who have never visited a battleship.

### THE DUKE OF RUTLAND'S DOUBLE WARNING.

Good use was made of the opportunity afforded to him at the quarterly meeting of the Middlesex Hospital by the Duke of Rutland last week. He emphasised the needs of civilian patients, and remarked, in hoping that they would always find them recognised, on the danger of the claims of the soldiers proving more "attractive" than the equally important claims of men and women out of khaki. The Duke of Rutland also paid a well-deserved tribute to the achievements of voluntary workers during this war, and, in allusion to the help given by the Volunteer Training Corps, declared that the way in which Volunteers had been treated by the Government had proved a cruel hardship to many men over military age. The movement had now received some official recognition, but if the Volunteers were to be adequately trained and maintained, the Government must make up their mind to recognise them on a financial as well as a friendly basis. The above is an instance of the way in which a capable chairman can make these quarterly meetings as important as they ought to be, through the medium of a good speech.

### A LONDON HOSPITAL MYSTERY.

Some interest has, it may plausibly be assumed, been excited among friends and patients of the London Hospital by a mysterious advertisement which has appeared in the "Agony" column of the *Times*. The advertisement asks the "lady occupants of the grey Rolls-Royce car" with limousine body that frequented the London Hospital during August and September 1915, or any of their friends, to communicate immediately with "Mr. L. A. Rawlie, bachelor," at a box address at the *Times* office. It goes on to say that "they will hear the true story of the past ten months, and will be amazed at the deception that has been and is being practised on them." Does this peculiar paragraph refer to a romance, a fraud, or a practical joke? Does it emanate from a medical student, a soldier patient, a nurse, a doctor, or an outsider? We do not know, but we feel certain that anyone who drives up to the "London" in a grey Rolls-Royce limousine car will be the subject of inquisitive glances if of nothing more.

### THE CITY AND THE ROYAL FREE HOSPITAL.

The Lord Mayor presided recently at a Mansion House meeting in support of the Royal Free Hospital. It was stated that a scheme has been started whereby this hospital may become a consultative centre for ante-natal and infant welfare clinics, a very appropriate arrangement in view of its position as a training school for women doctors, and of the share which women are taking in the management of these clinics. It is also proposed to provide additional accommodation for maternity as well as other patients on the site which, as already announced in The Hospital, has been given for this purpose. A statement has appeared in the Press that these extensions will absorb £200,000, towards which £5,000 is already subscribed; if this is so, the scheme is evidently a most ambitious one, and it would perhaps be well to adopt plans which will permit of its being carried out piecemeal as funds are obtained. At the meeting in question over £450 was secured; but £250 of this came from a single donor, so the success of the meeting, so far as immediate results are concerned, does not seem to have been overwhelming.

### MEDICAL GYMNASTICS AT HUDDERSFIELD.

Last year Huddersfield presented to the War Office an imposing military hospital at Royds Hall, which its citizens built and equipped at a cost of £24,000. This month the Freemasons of the Huddersfield and neighbouring lodges have added to it as their special gift a fully equipped gymnasium, which has now been opened. At the opening ceremony Colonel W. L. W. Marshall, R.A.M.C., the commanding officer, said that such a gymnasium had long been desirable, and, thanks to the donors and to the planning and equipment devised by Mr. K. F. Campbell, the borough engineer, the new department would be an immense advantage in treating stiff joints, impaired muscles, and nerve injuries. The gymnasium consists of a block of 40 feet by 20 feet, and its equipment includes wall bars, high plinth, low plinth, medical beam, medical stools, nautical wheel, peg post, a Sargent combination pulley machine, besides hand rings, wrist machine, and a stationary cycle exerciser. General Kenny, Northern Command, in replying for his wife, who performed the opening ceremony, described the institution as a model which receives interested visitors from all parts. With the masseur reinforced by medical gymnastics, the timely enlargement of general orthopædics in this country should be secure.

### THE SAMARITAN SOCIETY IN WAR-TIME.

The field in which the labours of the Hospital Samaritan Society usually lie must have become considerably contracted during the past eighteen months owing to the fact that so many hospital beds have been devoted to non-civilian cases, and that there has been a noticeable general decrease in many districts in the number of out-patients treated. But the field for this kind of work was always enormously out of proportion to the number of labourers. It is not surprising, bearing this in mind, to find a Samaritan Fund, even under the special war conditions instanced, working at full pressure. This is the case with the Samaritan Fund and Lady Almoner's Department of St. Thomas's Hospital, the report of which has been recently published. The department, as its title indicates, has the dual mission of preventing hospital abuse and providing means to ensure that the treatment afforded leads to as great permanent benefit as is possible in the circumstances of each case. In material assistance during 1915 1,057 patients were sent to convalescent homes at a total cost of £1,139, and a sum of £1,988 was expended by or through the fund towards the cost of instruments and appliances for patients. The care with which the circumstances of patients is inquired into is illustrated by the fact that 4,408 out-patients and casualty cases were refused treatment because they were able to pay private doctors, or were entitled and suited to the services of a panel doctor, or could be treated adequately by the Poor-Law authorities. The lady almoner at St. Thomas's invites and collects payments from patients towards the cost of their maintenance, etc.; in 1915 the out-patients contributed £209 and the in-patients £1,095. Insurance Committees paid on account of insured members the sum of £454.

### THE WOMAN DOCTOR IN THE DARK.

When the Lighting Regulations came into force last month the extended use of power electric torches, such as are used in theatres by the attendants who show late-comers into their seats, became inevitable. With the arrival of summer, under the Daylight Saving Act, one would have thought that they had become unnecessary. Nevertheless only last week a woman doctor was charged at Newcastle with using a flashlight. The witnesses included a special constable, and the defence was that, owing to the darkness, its user was in a hopeless position without a light. The defendant was not convicted but ordered to pay the cost of the witnesses. It seems strange that at the end of May darkness could descend so completely as to make a flashlight necessary. But weather varies; and even the Derby has been run in a snowstorm before now.

### A RESERVE STAFF.

At the recent opening of the new ward which has been built at the Penmore Isolation Hospital, Chesterfield, Mr. W. H. Edmunds pointed out that this was one of the few institutions the extension of which had been sanctioned by the Local Government Board. He also added the interesting point that, in respect of the administrative block, the committee were working towards the scheme of Dr. Barwise, under which a reserve staff of nurses would be maintained for loan, when emergency arose, to the various hospitals in the county. In the past, Mr. Edmunds explained, nurses had been obtained from other institutions, and the committee had been compelled "to pay through the nose" for them. Centralisation, here as elsewhere, is economical in principle; and the scheme associated with Dr. Barwise will be of interest to other counties which may be oppressed with the double task of having sufficient nurses to meet an emergency while avoiding unnecessary expense and overstaffing.

### PAPER-SAVING AT ST. MARY'S HOSPITAL.

The example set by Charing Cross Hospital has been followed, in spite of the deprecation of it which appeared in our correspondence columns last week, by the authorities of St. Mary's Hospital. The annual report contains forty-three instead of 116 pages, and the number of copies printed has been cut down from 3,500 to 500. As at Charing Cross, those subscribers who ask for a copy will receive one; but copies will not be sent broadcast to those who do not express the wish to have one. In spite of the contrary view, expressed last week by our correspondent, we adhere to our opinion that this paper-saving and money-saving device is praiseworthy and should be widely adopted. We hope other hospitals will follow it.

### SCOTTISH WAR HOSPITAL ACTIVITIES.

Sir Charles Cayzer has placed at the disposal of the war executive of the Scottish branch of the British Red Cross Society Ralston House and grounds, situated between Glasgow and Paisley, and is fitting up the mansion as a hospital for paralysed sailors and soldiers. This generous gift provides a haven of refuge for Scottish soldiers whose injuries have left them helpless. The hospital is so centrally situated as to be easily available to soldiers' relatives, and will command the best medical skill. Another recent development of Scottish Red Cross work which calls for mention is the provision of an *x*-ray ambulance car for Russia. The great distances which separate the Russian front and the hospitals make necessary an accurate diagnosis of injuries before the wounded are dispatched to the base. The Scottish *x*-ray car will be used in this connection, and will form a fitting addition to the Scottish Hospital ward at Petrograd.

### A HOSPITAL ARBITRATION.

A dispute having arisen between the burghs of Ardrossan and Saltcoats as to their respective share of the cost of maintaining their joint hospital—an expense formerly defrayed equally—the Local Government Board was asked to arbitrate. The Local Government Board has decided that Ardrossan must find nine-twentieths of the cost and Saltcoats eleven-twentieths. The saving to Ardrossan is £50 a year.

### THE AMBITIOUS GAZETTE.

The May-June number of the *Craigleith Hospital Chronicle* is a good instance of high aims in matter and presentment. The inset line drawings and illustrations, which by this process are permitted to be printed on the same paper in the text, are a real pleasure to the eye, and a genuine decoration to the pages, and remind eyes weary of the glitter and unpleasant touch of art paper (required for half-tone blocks) of the ideal age of illustration, when wood-cuts were the rule, or when, as in the eighteen-sixties, line drawings were the staple way of illustrating. The story of the Scottish regiments, Number 8 of this series, the account of the regalia of Scotland, the article on Easter in Russia, charmingly illustrated, the tale of the new recruit with the wonderful name: all these show the high standard which this magazine has set and maintained, and reminds us that chaff and jokes, good in their way, have formidable rivals in articles which attempt something more than mere amusement.

### A REWARD FOR COTTAGE HOSPITAL TRAINING.

The ambitious nurse generally joins the staff of a cottage hospital only in the hope of filling up time till she can obtain more varied opportunities in one of the larger institutions. But since the war has led to the upspringing of a number of convalescent homes for soldiers, where the nursing is light and a knowledge of country housekeeping a principal asset, the post of matron of a convalescent home for soldiers has provided a new field for the peculiar experience afforded by a nurse who has had the experience that a cottage hospital training can give her. It is true that the matrons of these convalescent homes have had wider experience as well; but there are many nurses who, for one reason or another, count their cottage hospital experience as the chief event in their record, and for these, other things being equal, the post of matron at a military convalescent home offers congenial as well as suitable work.

### A NEW TUBERCULOSIS ORDER.

The Local Government Board have recently issued a new General Order dealing with the notification of tuberculosis, which will be known as the "Public Health (Tuberculosis) Regulations, 1916." The Order, which is to remain in force during the continuation of the present war, has for its object the prevention of the enlistment into the Army of men suffering from tuberculosis. Medical officers of health will in future be required to furnish to the Army Council particulars of all male persons between the ages of sixteen and forty-five who have been notified since February 1, 1913, as suffering from tuberculosis. In the Circular accompanying the Order addressed to Town Councils, Metropolitan Borough Councils, Urban and Rural District Councils the Board express their confidence that the local authorities and their officers will readily co-operate in supplying the required particulars. Past experience has shown the unwisdom of admitting a tuberculous individual, no matter in what stage of the disease he may be, into the Army. In spite of the fact that a few cases of incipient phthisis have been cured after "joining up," the best medical authorities are in favour of the total exclusion of all early and doubtfully tuberculous subjects.

### ASSISTANCE FOR TUBERCULOUS SOLDIERS.

The treatment of men discharged from the Army on account of tuberculosis appears to be exercising the minds of several of the local authorities at the present moment. The Local Government Board have approached certain of the Municipal Health Committees and the Town Councils to ascertain whether the latter would be willing to contribute towards the cost of treatment in the case of uninsured soldiers discharged from the Army as being tuberculous. For example, it was reported that a Yarmouth man was being treated in a sanatorium in Kent at a cost of 35s. a week, and that the Corporation of the Norfolk town was asked to defray half this amount. In the case of Bournemouth we understand that the medical officer of health has reported advising that men who have been discharged in this manner from the Army should be dealt with under the municipal scheme which is free to all the community. In this instance the Public Health Committee agreed in principle with the medical officer's advice. It seems reasonable that a proportion, say, one-half, as recommended by the Treasury, of the cost of the treatment of these unfortunate men should be defrayed from the Exchequer maintenance grant in aid of such schemes, while the local authority should be called upon to provide the remainder.

### THE METROPOLITAN ASYLUMS BOARD AND ITS EMPLOYEES.

It is to be hoped that the Metropolitan Asylums Board has surmounted the temporary trouble with its employees, who presented a petition praying for the redress of certain grievances under which they were suffering in the various asylums. The call to the Colours has seriously depleted the staffs, with the result that those remaining have had to perform heavy additional duties, and members of the permanent staff have had to fill the posts of the higher grade officials. Hitherto they have received no extra remuneration for these extraneous duties, but the managers have now happily decided that where it is shown that additional responsibilities are undertaken or extra work is performed, a grant may be made in recognition of the officer's exertions. A grievance of long standing is the payment for meals to officers when they have their weekly day of rest. Hitherto the managers have not been favourable to making any allowance, but now they have decided to pay the male married employees living outside the institutions, yet having their meals inside, for the three meals a day when off duty. By granting these two concessions it is understood that the trouble is over for the present, and that the employees will waive their chief claim for a large increase in the scale of pay, which the managers maintain is entirely out of the question during the continuance of the war.

### NURSES AND THE NATIONAL POOR-LAW OFFICERS' ASSOCIATION.

The Organisation Sub-Committee of the National Poor-Law Officers' Association reports that "up to the present all efforts to enrol Metropolitan Poor-Law nurses in our ranks have met with the determined hostility of those best able to influence the nursing staffs." Commenting upon this, a writer in the *Poor-Law Officers' Journal* asks what is the cause of this hostility. We referred to the matter some little time back, and indicated the answer. Most of those best able to influence the nursing staffs in the Metropolitan Poor-Law infirmaries are convinced that it is desirable that the infirmaries should be removed from the control of Poor-Law Guardians, and that in the non-separated infirmaries the workhouse master should have no administrative powers in the sick wards. The National Poor-Law Officers' Association is in both respects the champion of the existing order of things. Hence the main cause of the hostility to the Association.

### THE ROYAL COMMISSION ON VENEREAL DISEASES.

Dr. Baly, medical superintendent of Lambeth Infirmary, has presented to the Lambeth Board of Guardians a report dealing with the treatment of venereal diseases in his infirmary. This is highly important in view of the report of the Royal Commission on Venereal Diseases. Dr. Baly states that patients suffering from syphilis (unless there are contra-indications) are given three injections, intravenously of galyl (a French preparation similar to salvarsan), which is followed by six courses of intra-muscular injections of metallic mercury in the form of grey oil; each course consists of six injections at intervals of one week; an interval of six weeks is allowed to elapse between each course. All patients are kept in the infirmary as long as any syphilitic lesions are present, and, when possible, to the end of the first mercurial course. Since this routine had been adopted there had not been any case of relapse returning to the infirmary, and there had been two cases of re-infection. Experience so far showed that 60 per cent. of the women, but only a small percentage of the men, return regularly for treatment.

### THE DETENTION OF CASES.

Dr. Baly adds that on one or two occasions in the past the Board had detained patients under Section 22 of the Poor Law Amendment Act, 1867, but no cases had lately arisen in which this course was desirable, and doubts were expressed in Section 177 of the report under discussion as to the legality of this procedure. A Wassermann reaction was obtained for purposes of diagnosis in doubtful cases only, and not as a guide to treatment, owing to the expense, which would only be justified if these reactions could be performed in the infirmary laboratory with the aid of an expert pathologist. The cost of these reactions amounted to £50 during the year, and 220 doses of galyl had been given, costing £113, making a total of £163 in all. Any patient applying at the infirmary suffering from a venereal disease had been admitted as "urgently in need of medical treatment," without a relieving officer's order, and he recommended that this procedure should be made known as widely as possible, and, further, that the disease from which these patients were suffering should be treated as strictly confidential. In order to carry this out effectively it would be necessary to treat *all* cases in the infirmary as confidential, and to give information as to the disease from which patients were suffering only when it had some direct bearing on the relief required, as in recommending a patient suffering from tuberculosis for a sanatorium. The Guardians have adopted the suggestions contained in Dr. Baly's report.

### THE BELFAST GUARDIANS AND THEIR MEDICAL OFFICERS.

The dispute between the Belfast Guardians and their medical officers as to the remuneration payable in respect of military patients in the Poor-Law infirmary has at last been settled. We referred to the matter in a recent number of The Hospital, and criticised the position taken up by some of the Guardians, who maintained that, because military patients were attended gratuitously by the medical staff of voluntary hospitals, the doctors of the infirmary were acting unpatriotically in demanding payment for similar services. A special committee was appointed to confer with the medical staff upon the matter. The evidence placed before this committee convinced the members that the infirmary, so far as the military patients were concerned, was a military hospital, and that the visiting medical officers were entitled to War Office pay—viz., one guinea per day, irrespective of the number of military patients treated. The medical staff was, however, prepared to accept a reduced scale of payment at the rate of 3s. per week per patient, subject to a maximum rate of seven guineas per week. The committee recommended this scale to the Board for adoption, and the recommendation was approved with only five dissentients. As the War Office are paying one guinea a week for each military patient, and as it is admitted that this exceeds the maintenance rate by at least 3s., the Guardians cannot lose by the arrangement, and if the whole of their available accommodation is required, they will gain considerably by it.

### CONTRACTS BETWEEN GUARDIANS AND MEDICAL OFFICERS.

The Shaftesbury Board of Guardians recently approached the Local Government Board to obtain sanction for a new contract with the medical officer of their workhouse, under which it was proposed to reduce his salary from £50 to £40, and to make his appointment terminable by notice. In their reply the Board state that they are not prepared to require the medical officer to accept a reduced salary unless it is shown that the duties have diminished substantially since the present salary was fixed, and that the remuneration is now excessive in view of the services performed. They point out that the appointment of a medical officer can only be determined by the Local Government Board, and that provisions as to notices contained in a contract would have no effect as regards a medical officer's tenure of office. In The Hospital of August 14, 1915, we drew attention to the fact that many Boards of Guardians had made contracts with their medical officers making their appointments terminable by notice, and pointed out that the Local Government Board had ruled that an agreement of this kind could not be enforced. It is important that all medical officers should understand that the fixity of tenure in their office laid down in the Consolidated Orders cannot be abrogated by any contract with a Board of Guardians. The Shaftesbury Guardians resent the position taken up by the Local Government Board, and threaten to resign if they cannot have their own way. This threat is hardly likely to lead the Board to alter its decision.

### THE MEDICAL OFFICER'S SALARY AT DRIFFIELD.

The dispute between the Driffield Board of Guardians and their medical officer, to which we drew attention in a recent number of The Hospital, has advanced another stage. We referred to the fact that the medical officer had appealed to the Local Government Board against the proposed reduction of his salary from £10 to £5. As we anticipated, the Board has now informed the Guardians that it does not think the medical officer's salary could be reduced as suggested. The Guardians have referred the correspondence to the house committee for consideration. The Guardians have already expressed regret that they adopted the resolution to reduce the medical officer's salary without consulting him, and if they are wise they will now rescind the resolution and let the matter drop.

### THIS WEEK'S DRUG MARKET.

The downward tendency in prices which has been noticeable during the past few weeks is not so much in evidence this week; but the upward movement in the values of those drugs which until recently had been continually advancing is, generally speaking, at a standstill. A large business has again been done in hexamine, and the price tends upward. There is a large demand for pilocarpine hydrochloride which is difficult to meet. Phenacetin is substantially dearer, and it is not improbable that prices will advance still further. Adrenine continues very scarce, but the demand has fallen off considerably. Trional and barbitone are dearer in consequence of increasing scarcity. There is a strong demand for senega root, and a good business has been done. Practically no business is being done in cod-liver oil, owing to the high prices asked. Exceedingly high figures are quoted for dandelion root, colchicum seed, and belladonna root. Lycopodium is difficult to obtain. A good business is being done in benzoic acid and sodium benzoate at high prices. Tartaric acid and citric acid tend downwards in price. Oil of cloves is dearer. There is a distinctly lower tendency in the values of salicylic acid and salicylate of soda, this being due to the fact that British manufacturers of these products are continuing to increase their output, and have brought the quality of salicylates almost up to the standard of American imports.

